# Ten-year outcomes of surgical aortic valve replacement with a contemporary supra-annular porcine valve in a Medicare population

**DOI:** 10.1016/j.xjon.2022.08.002

**Published:** 2022-08-17

**Authors:** Robert J. Wiechmann, Leonard Y. Lee, Yang Yu, Julie B. Prillinger, Dan Gutfinger, Bradford Blakeman

**Affiliations:** a Mayo Clinic Health System, Cardiothoracic Surgery, Eau Claire, Wis; b Division of Cardiothoracic Surgery, Rutgers Robert Wood Johnson Medical School, New Brunswick, NJ; c Abbott, Santa Clara, Calif; d UP Health System–Marquette, Cardiovascular Surgery, Marquette, Mich

**Keywords:** aortic valve replacement, durability, heart failure, porcine valve, survival, CABG, coronary artery bypass grafting, CI, confidence interval, CMS, Centers for Medicare & Medicaid Service, FFS, fee-for-service, HF, heart failure, HR, hazard ratio, ICD-9, International Classification of Diseases, Ninth Revision, ICD-10, International Classification of Diseases, Tenth Revision, LVAD, left ventricular assist device, SAVR, surgical aortic valve replacement, TAVI, transcatheter aortic valve implantation, VIV, valve-in-valve

## Abstract

**Objective:**

Bioprosthetic surgical aortic valve replacement remains an important treatment option in the era of transcatheter interventions. Real-world outcomes are not well characterized because of limited prospective follow-up studies. We present the 10-year clinical outcomes of Medicare beneficiaries undergoing surgical aortic valve replacement with a contemporary supra-annular porcine valve.

**Methods:**

This is a single-arm observational study using Medicare fee-for-service claims data. De-identified patients undergoing surgical aortic valve replacement with the Epic Supra valve (Abbott) in the United States between January 1, 2008, and December 31, 2019, were selected by International Classification of Diseases 9^th^ and 10^th^ Revision procedure codes and then linked to a manufacturer device tracking database. All-cause mortality, heart failure rehospitalization, and aortic valve reintervention (surgical or transcatheter valve-in-valve) were evaluated at 10 years using the Kaplan–Meier method.

**Results:**

Among 272,591 Medicare beneficiaries undergoing surgical aortic valve replacement during the study period, 11,685 received the Epic Supra valve, of whom 51.6% (6029) had underlying heart failure. Mean age was 76 ± 7 years. Survival at 10 years in patients without preoperative heart failure was 43.5% (95% confidence interval, 41.8-45.2) compared with 24.1% (95% confidence interval, 22.6-25.5) for patients with heart failure (*P* < .001). The 10-year freedom from heart failure rehospitalization was 64.0% (95% confidence interval, 62.6-65.3). Freedom from aortic valve reintervention was 94.6% (95% confidence interval, 93.8-95.3) at 10 years.

**Conclusions:**

This real-world nationwide study of US Medicare beneficiaries receiving the Epic Supra valve demonstrates more than 94% freedom from all-cause valve reintervention and 64% freedom from heart failure rehospitalization at 10 years postimplant. Long-term survival and heart failure rehospitalization in this population with aortic valve disease undergoing surgical aortic valve replacement were found to be impacted by underlying heart failure.

## Graphical abstract

**Figure:**
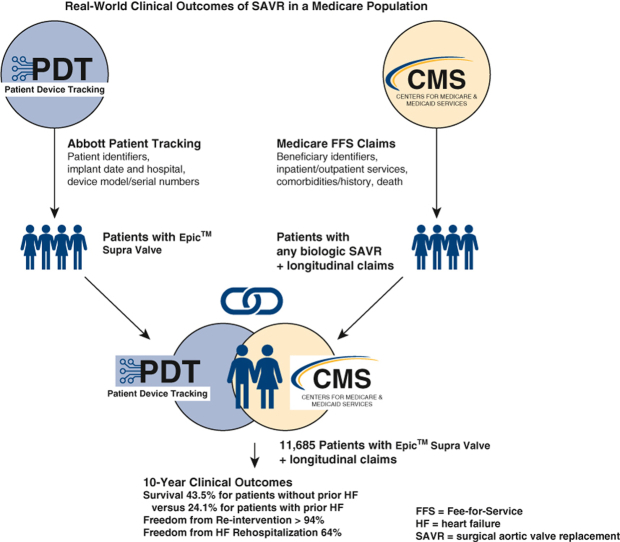

**Figure:**
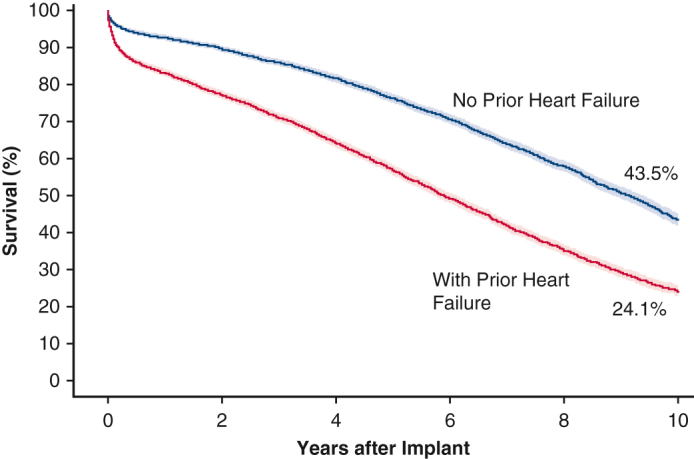
Survival of SAVR in patients with and without HF.

**Central Message**

Clinical outcomes of SAVR in a Medicare population are impacted by underlying comorbidities and the need for concomitant cardiac procedures.

**Perspective**

SAVR remains an important treatment option for the Medicare population in the era of transcatheter valve interventions. Clinical outcomes are impacted most by preoperative HF and renal failure and the need for concomitant CABG or other cardiac valve surgery.

Over the past decade, there has been tremendous growth in the adoption of transcatheter aortic valve implantation (TAVI) as lower-risk patients became eligible for treatment and as continuous improvements were made in clinical outcomes and the available TAVI technology.^1^ Although in recent years more patients within the Medicare population are undergoing TAVI, bioprosthetic surgical aortic valve replacement (SAVR) continues to remain an important treatment option for patients with aortic valve disease, especially those who require additional concomitant cardiac procedures.^2^, ^3^, ^4^ A recent analysis of Medicare data showed that the acute clinical outcomes with TAVI have continued to improve over the last decade, whereas the clinical outcomes with SAVR have remained relatively unchanged considering SAVR is a more mature procedure.^4^ Gaining insights into the long-term clinical outcomes of SAVR and the impact of underlying comorbidities and concomitant procedures would be informative to surgeons counseling patients who need an aortic valve replacement. The objective of this study is to assess the 10-year clinical outcomes of Medicare beneficiaries undergoing SAVR with the Epic Supra valve (Abbott, Santa Clara, Calif) in the era of transcatheter valve interventions.

## Materials and Methods

### Data Sources

This was a single-arm retrospective observational study using real-world data^5^ derived from the Centers for Medicare & Medicaid Services (CMS) administrative claims data linked to a manufacturer device registration database (Abbott). Patient baseline characteristics (age, gender, race, and comorbidities), SAVR procedure date and hospital, and outcomes were identified from CMS fee-for-service (FFS) claims files. The manufacturer device registration database was used to determine the specific valve model and size implanted (Table E1). The use of CMS records linked with manufacturer device registration data was approved through a data use agreement with CMS (RSCH-2020-54878). The study protocol was approved on June 25, 2019, by Western Institutional Review Board (Institutional Review Board Study Number 1261727) with a waiver of informed patient consent because the study was a minimal risk database analysis.

### Study Population

All Medicare beneficiaries undergoing SAVR with a bioprosthetic heart valve from January 1, 2008, to December 31, 2019, were identified using International Classification of Diseases, Ninth Revision (ICD-9) and Tenth Revision (ICD-10) codes (Table E2). Beneficiaries were excluded if they did not have continuous coverage in Medicare Part A and Part B for at least 12 months preceding the SAVR admission to allow for characterization of baseline comorbidities, using the Charlson and Elixhauser comorbidity algorithms^6^ (Table E3). Manufacturer device registration data were linked to the CMS records using the probabilistic method with a combination of secondary patient identifiers (implant date, date of birth, gender, and implant hospital if available) as previously described.^7^^,^^8^ For patients who underwent multiple SAVR procedures as identified in the CMS claims data, the index event was defined as the first hospitalization during which the Epic Supra valve (Abbott) was implanted. Beneficiaries with a diagnosis of endocarditis during the index hospitalization were identified using ICD-9 and ICD-10 diagnosis codes (Table E3) and were excluded from the analysis. The study cohort was followed from the date of implant until death, the end of Medicare FFS enrollment, the start of Medicare Advantage program enrollment, or the end of data availability (December 31, 2020), whichever came first.

The presence of concomitant procedures including coronary artery bypass grafting (CABG) or other cardiac valve surgery (surgical mitral/tricuspid/pulmonary valve repair or replacement) during the SAVR hospitalization was identified using ICD-9 and ICD-10 procedure codes (Table E3). A subgroup analysis on patients with isolated SAVR that excludes patients who underwent a concomitant CABG or other cardiac valve surgery was performed. The isolated SAVR subgroup also excluded patients with a redo SAVR, which was defined as having a prior TAVI or a prior surgical aortic valve repair or replacement. The use of acute mechanical circulatory support or a diagnosis of cardiogenic shock during the index hospitalization was used as a proxy for emergency SAVR and was also excluded from the isolated SAVR subgroup (Table E3).

The Epic Supra valve (Abbott) is a bioprosthetic heart valve that incorporates a triple composite design and is manufactured from selected porcine valve cusps that are matched for optimum leaflet coaptation and hemodynamics.^9^, ^10^, ^11^ The Epic Supra valve is processed with an ethanol-based anti-calcification treatment (Linx AC) that in animal studies prevented calcification.^12^ The Epic Supra valve was approved for commercial use in the United States in November 2007.

### Clinical Outcomes

The study objective was to evaluate long-term clinical outcomes in a real-world setting including survival, freedom from heart failure (HF) rehospitalization, and freedom from reintervention at 10 years post-SAVR. Date of death was acquired from the CMS Master Beneficiary Summary File and was used to calculate the survival time and all-cause mortality. Operative mortality was defined as any mortality encountered through 30 days postimplant or during the index hospitalization, whichever occurred later. A HF rehospitalization was identified as any inpatient encounter after SAVR with a primary diagnosis of HF (Table E3), as previously described.^13^ Aortic valve reintervention was defined as including a subsequent surgical valve replacement with a bioprosthetic or mechanical heart valve or a transcatheter valve-in-valve (VIV) implantation within the Epic Supra valve (Table E4). A terminal end point was evaluated at 10 years postimplant, which included left ventricular assist device (LVAD) implant or heart transplant. The valve reintervention end point and terminal end point were identified using the corresponding ICD-9 and ICD-10 procedure codes (Table E4) in the CMS inpatient claims after the index SAVR hospitalization.

### Statistical Analysis

Patients' baseline demographics and operative details were summarized using standard statistics, such as mean (standard deviation) and count (percentage) as appropriate. Operative mortality and the terminal end point were reported in crude rates as the number of events divided by the total number of patients in the applicable cohort. Cumulative incidence rates per person-year were presented for all-cause rehospitalization and HF rehospitalization. Survival curves and estimates for 10-year survival, freedom from HF rehospitalization, and freedom from reintervention were generated using the Kaplan–Meier method. Differences between curves were compared using the log-rank test. Cox proportional hazards models were applied to all-cause mortality and HF rehospitalization outcomes in subgroup analyses. Independent predictors of 10-year mortality and HF rehospitalization were determined using multivariable Cox regression models. All *P* values were based on 2-sided tests. All analyses were performed using SAS 9.4 (SAS Institute, Inc).

## Results

### Study Cohort

**Figure 1:**
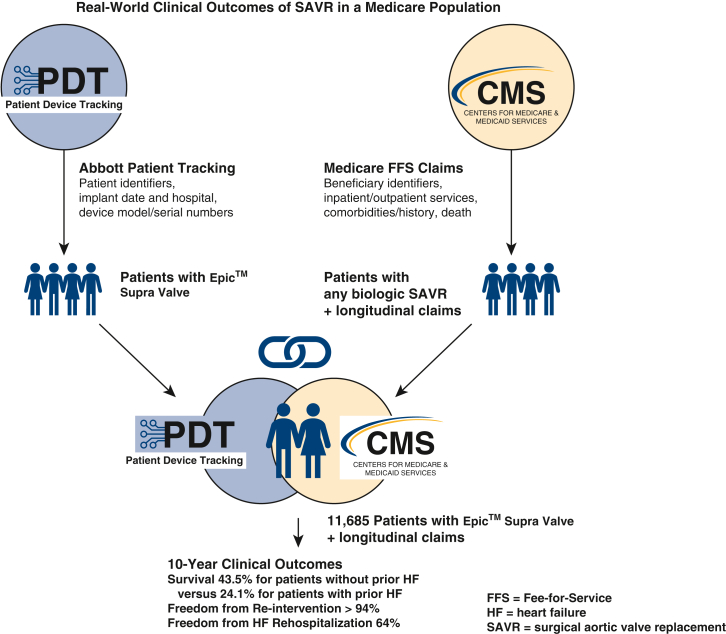
Real-world clinical outcomes of SAVR with the Epic Supra Valve (Abbott) in a Medicare Population. *SAVR*, Surgical aortic valve replacement; *PDT*, patient device tracking; *CMS*, Center for Medicare & Medicaid Services; *FFS*, fee-for-service; *HF*, heart failure.

**Figure E1:**
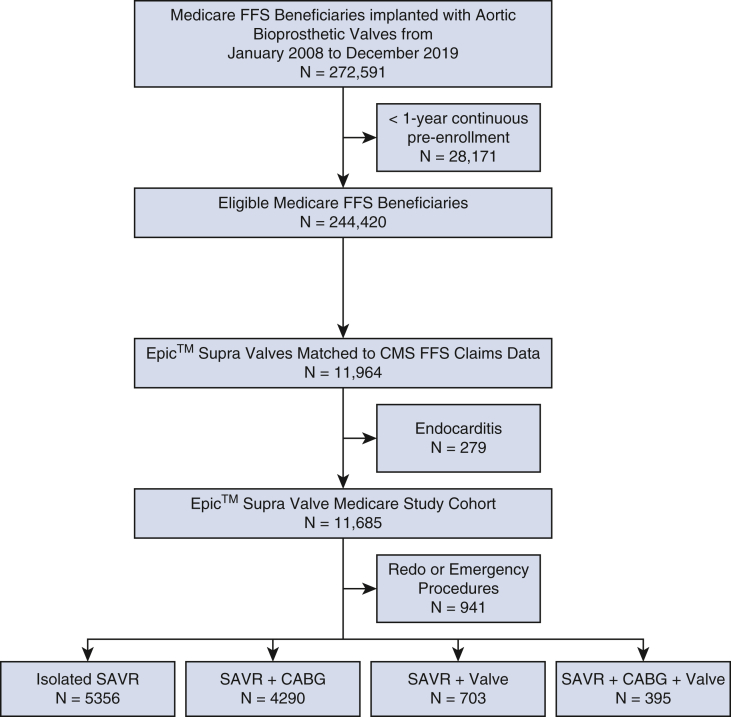
Study cohort diagram. *FFS*, Fee-for-service; *CMS*, Center for Medicare & Medicaid Services; *SAVR*, surgical aortic valve replacement; *CABG*, coronary artery bypass grafting; *VALVE*, other cardiac valve repair or replacement (mitral, tricuspid, or pulmonary).

Of 244,420 Medicare FFS beneficiaries who underwent a SAVR during the study period and had at least 1 year of continuous enrollment in Medicare FFS before the index SAVR, 11,964 received the Epic Supra valve according to the manufacturer device registration database. After excluding 279 beneficiaries who had a diagnosis of endocarditis at the index admission, all outcomes were derived from the remaining 11,685 patients (Figures 1 and E1). The Epic Supra valve implants were performed at more than 650 medical centers across all states of the United States.

Baseline patient demographics and medical history are presented in Table 1. The mean age of the study cohort was 76.3 ± 7.1 years with an average follow-up of 5.2 ± 3.3 years post-SAVR. Women represented 39.5% (n = 4615) of the study cohort, and 93.7% (n = 10,946) were White. The baseline age, gender, and race for the study cohort were similar to the baseline demographics of patients with bioprosthetic SAVR who were not linked to the manufacturer device registration database. The Medicare study cohort was characterized by a high burden of comorbidities, including 51.6% (n = 6029) with HF, 33.6% (n = 3924) with atrial fibrillation, and 25.8% (n = 3016) with renal failure, including 2.0% (n = 229) on dialysis.

**Table 1:** Patient demographics and medical history

Variable	N = 11,685
Age at implant (y)	76.3 ± 7.1
Gender (female)	4615 (39.5%)
Race (White)	10,946 (93.7%)
Medical history	
Hypertension	11,047 (94.5%)
Cerebrovascular disease	7103 (60.8%)
Peripheral vascular disease	6839 (58.5%)
Chronic pulmonary disease	6499 (55.6%)
HF	6029 (51.6%)
Diabetes	5699 (48.8%)
Atrial fibrillation	3924 (33.6%)
Renal failure	3016 (25.8%)
Renal failure with dialysis	229 (2.0%)
Previous myocardial infarction	2635 (22.6%)
Obesity	2619 (22.4%)
Coagulopathy	1744 (14.9%)
Liver disease	1574 (13.5%)
Prior HFH (all time)	1282 (11.0%)
No. of prior HFHs within 1 y of SAVR	
0	10,691 (91.5%)
1	801 (6.9%)
2	122 (1.0%)
3+	71 (0.6%)

*HF*, Heart failure; *HFH*, heart failure hospitalization; *SAVR*, surgical aortic valve replacement.

Table 2 shows the SAVR operative details. More than 60% of the patients underwent SAVR between 2008 and 2011, before the commercial introduction of TAVI in the United States and other options for bioprosthetic SAVR. During the index hospitalization, 44.9% (n = 5243) of the study cohort underwent a concomitant CABG procedure and 11.2% (n = 1307) had other concomitant cardiac valve surgery. The proportion of isolated SAVR versus the overall number of SAVR cases remained relatively stable over the study period (Table E5). The 2 most commonly used valve sizes for the Epic Supra valve were 23 mm (34.0%, n = 3969) and 21 mm (29.9%, n = 3497). The overall hospital length of stay for the index hospitalization of SAVR was 10.6 ± 8.8 days.

**Table 2:** Operative details

Variable	N = 11,685
Implanted valve size, mm	
19	1349 (11.5%)
21	3497 (29.9%)
23	3969 (34.0%)
25	2218 (19.0%)
27	652 (5.6%)
Concomitant procedures	
CABG	5243 (44.9%)
Other cardiac valve repair or replacement (mitral, tricuspid, or pulmonary)	1307 (11.2%)
Prior SAVR, surgical aortic valve repair, or prior TAVI	44 (0.4%)
Emergency SAVR	903 (7.7%)
Nonemergency and non-redo procedures	10,744 (91.9%)
Isolated SAVR	5356 (45.8%)
SAVR + CABG	4290 (36.7%)
SAVR + valve repair or replacement (mitral/tricuspid/pulmonary)	703 (6.0%)
SAVR + CABG + valve repair or replacement (mitral/tricuspid/pulmonary)	395 (3.4%)
Hospital length of stay (d)	10.6 ± 8.8
Year of implant	
Implant year (2008-2009)	2924 (25.0%)
Implant year (2010-2011)	4245 (36.3%)
Implant year (2012-2013)	1928 (16.5%)
Implant year (2014-2015)	1357 (11.6%)
Implant year (2016-2017)	750 (6.4%)
Implant year (2018-2019)	481 (4.1%)

*CABG*, Coronary artery bypass grafting; *SAVR*, surgical aortic valve replacement; *TAVI*, transcatheter aortic valve implantation.

### Operative Mortality

**Figure E2:**
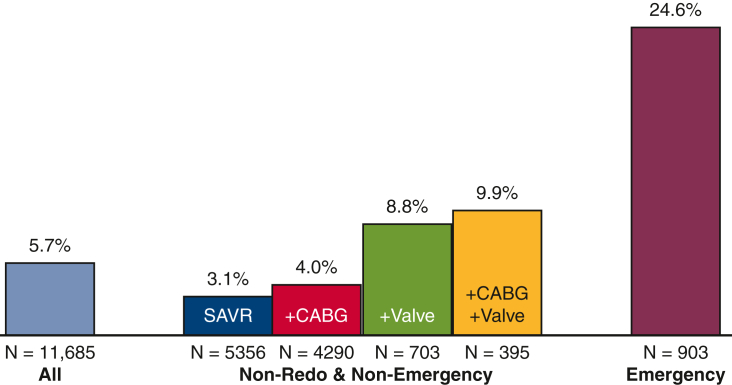
Operative mortality stratified by SAVR subgroups with concomitant procedures and emergency status. *SAVR*, Surgical aortic valve replacement; *CABG*, coronary artery bypass grafting; *VALVE*, other cardiac valve repair or replacement (mitral, tricuspid, or pulmonary).

Operative mortality for the overall Medicare cohort was 5.7%. Within various subgroups, patients undergoing isolated SAVR had the lowest mortality (3.1%) followed by those with concomitant procedures: 4.0% in patients with concomitant CABG, 8.8% in patients with other cardiac valve surgery, and 9.9% in patients with concomitant CABG and other cardiac valve surgery (Figure E2). Operative mortality was the highest for patients undergoing emergency SAVR at 24.6%.

### Long-Term Outcomes

During the 10 years after SAVR, fewer than 11 patients (<0.1%) met the terminal end point of LVAD implant or heart transplant. The all-cause rehospitalization rate was 0.69 per person-year and HF rehospitalization was 0.08 per person-year.

**Figure 2:**
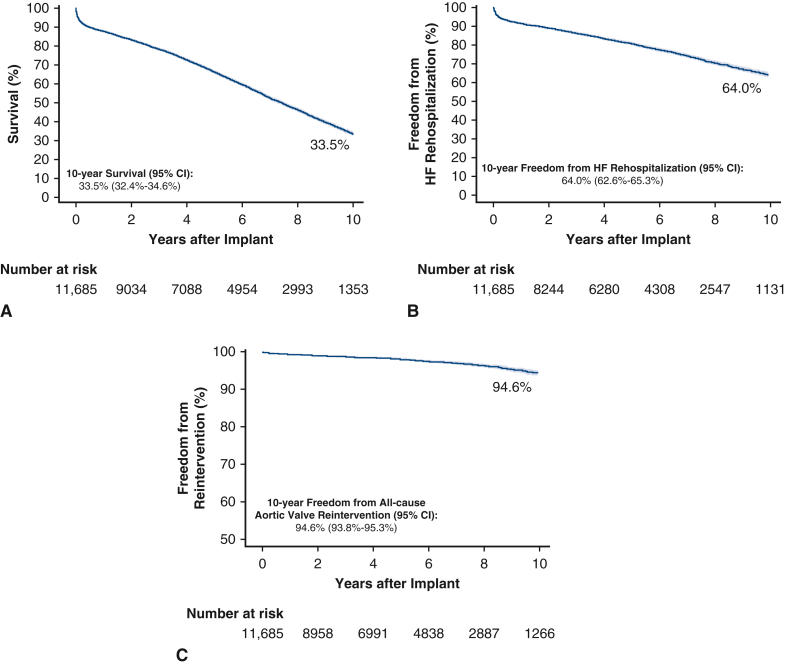
A, Kaplan–Meier 10-year survival. B, Kaplan–Meier 10-year freedom from HF rehospitalization. C, Kaplan–Meier 10-year freedom from all-cause aortic valve reintervention (reintervention includes repeat SAVR or a transcatheter VIV intervention, where patients were also censored at left ventricular assist device implant or heart transplantation). *CI*, Confidence interval; *HF*, heart failure.

Long-term clinical outcomes are presented in Figure 2. The 10-year Kaplan–Meier estimates of survival and freedom from HF rehospitalization were 33.5% (95% confidence interval [CI], 32.4-34.6) and 64.0% (95% CI, 62.6-65.3), respectively (Figure 2, *A* and *B*). At 10 years postimplant, there were 185 repeat SAVR procedures and 95 transcatheter VIV procedures, and the freedom from all-cause reintervention was 94.6% (95% CI, 93.8-95.3) (Figure 2, *C*). When considering the competing risk of death, the 10-year cumulative incidence rate of reintervention was 3.2% (95% CI, 2.8-3.6). Among the 185 repeat SAVR procedures, there were 59 cases with a diagnosis of endocarditis at the time of reoperation, whereas none of the 95 VIV procedures were associated with a diagnosis of endocarditis.

### Subgroup Analyses

**Figure 3:**
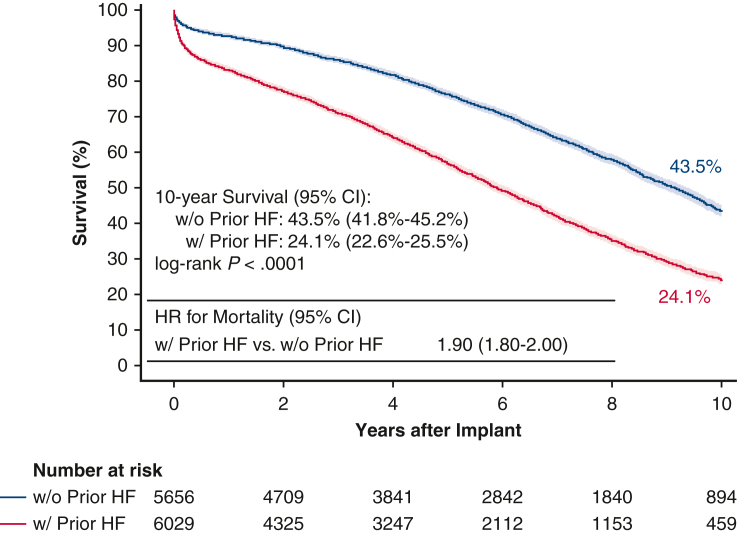
Kaplan–Meier 10-year survival stratified by baseline HF. *CI*, Confidence interval; *HF*, heart failure; *HR*, hazard ratio.

**Figure 4:**
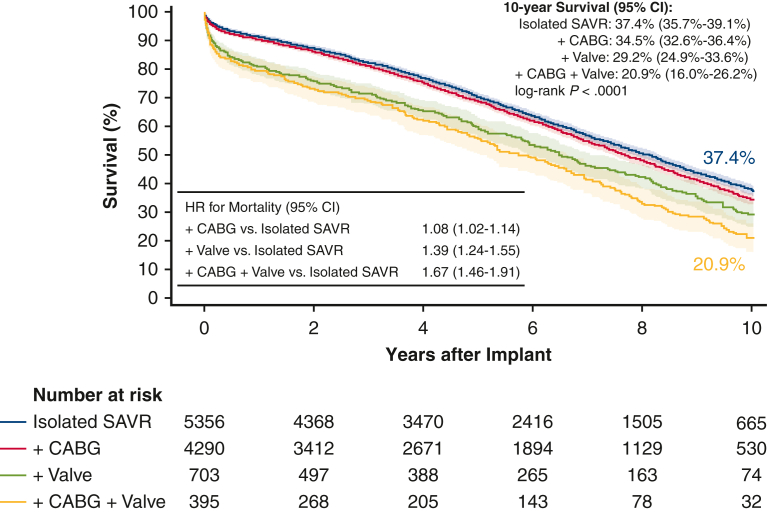
Kaplan–Meier 10-year survival stratified by SAVR subgroups with concomitant procedures. Isolated SAVR shown in *blue*. SAVR with concomitant CABG shown in *red*. SAVR with concomitant other cardiac valve repair or replacement (mitral, tricuspid, or pulmonary) shown in *green*. SAVR with concomitant CABG and other cardiac valve repair or replacement shown in *yellow*. *CI*, Confidence interval; *SAVR*, surgical aortic valve replacement; *CABG*, coronary artery bypass grafting; *VALVE*, other cardiac valve repair or replacement (mitral, tricuspid, or pulmonary); *HR*, hazard ratio.

**Figure E3:**
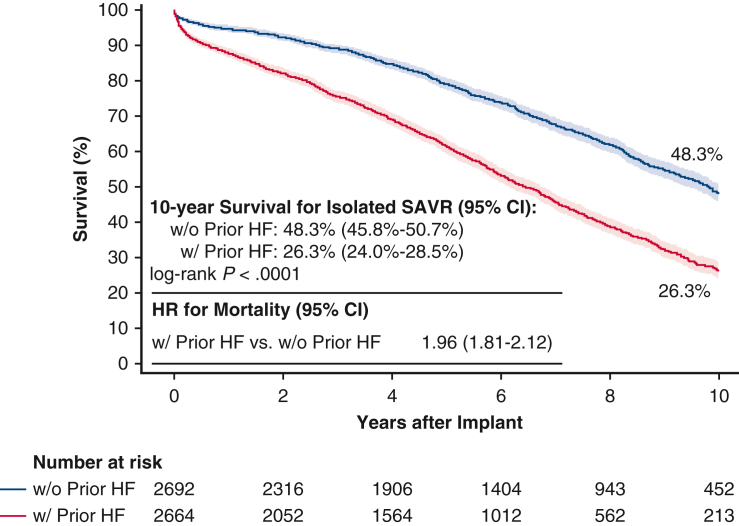
Kaplan–Meier 10-year survival for subgroup of isolated SAVR stratified by baseline HF. *SAVR*, Surgical aortic valve replacement; *CI*, confidence interval; *HF*, heart failure; *HR*, hazard ratio.

SAVR patients without a preoperative diagnosis of HF were associated with better survival at 10 years compared with those with a preoperative diagnosis of HF (43.5% vs 24.1%, log-rank *P* < .0001) (Figure 3). The magnitude of this trend in the 10-year survival in patients without versus with preoperative HF increased slightly in the subgroup of isolated SAVR (48.3% vs 26.3%, log-rank *P* < .0001; Figure E3). Consistent with the trend in operative mortality, the 10-year Kaplan–Meier survival among isolated SAVR patients was 37.4% (95% CI, 35.7-39.1), and was sequentially lower at 34.5% (95% CI, 32.6-36.4) for those with concomitant CABG; 29.2% (95% CI, 24.9-33.6) for those with concomitant other cardiac valve surgery; and 20.9% (95% CI, 16.0-26.2) for those with both concomitant CABG and other cardiac valve surgery (log-rank *P* < .0001) (Figure 4).

**Figure E4:**
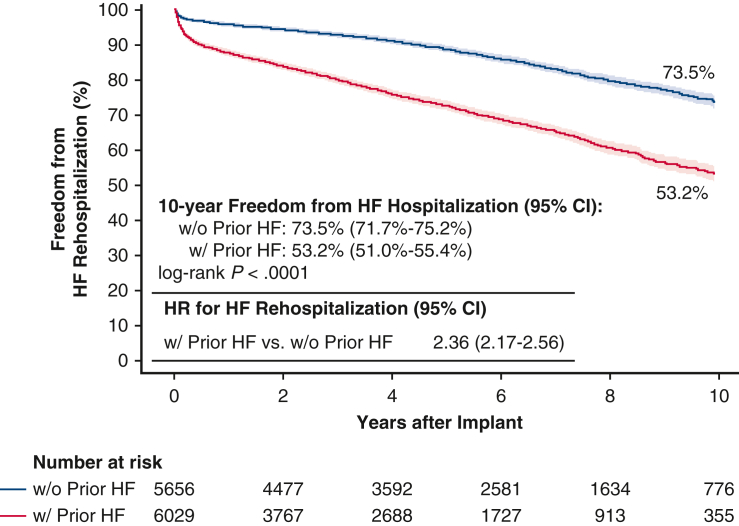
Kaplan–Meier 10-year freedom from HF rehospitalization stratified by baseline HF. *HF*, Heart failure; *CI*, confidence interval; *HR*, hazard ratio.

**Figure E5:**
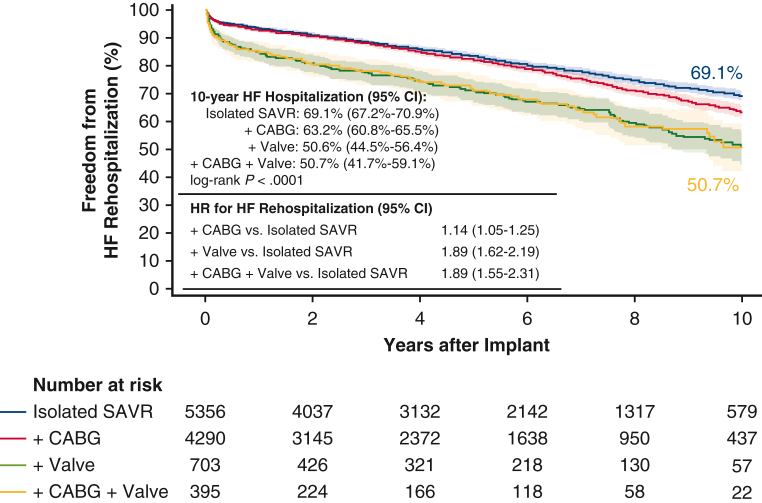
Kaplan–Meier 10-year freedom from HF rehospitalization stratified by SAVR subgroups with concomitant procedures. Isolated SAVR shown in *blue*. SAVR with concomitant CABG shown in *red*. SAVR with concomitant other cardiac valve repair or replacement (mitral, tricuspid, or pulmonary) shown in *green*. SAVR with concomitant CABG and other cardiac valve repair or replacement shown in *yellow*. *HF*, Heart failure; *CI*, confidence interval; *SAVR*, surgical aortic valve replacement; *CABG*, coronary artery bypass grafting; *HR*, hazard ratio; *VALVE*, other cardiac valve repair or replacement (mitral, tricuspid, or pulmonary).

Similar trends were observed for the 10-year freedom from HF rehospitalization (Figures E4 and E5). In the subgroup of isolated SAVR, the freedom from HF rehospitalization at 5 years and 10 years postimplant was 83.2% (95% CI, 82.1-84.3) and 69.1% (95% CI, 67.2-70.9), respectively (Figure E5).

**Figure 5:**
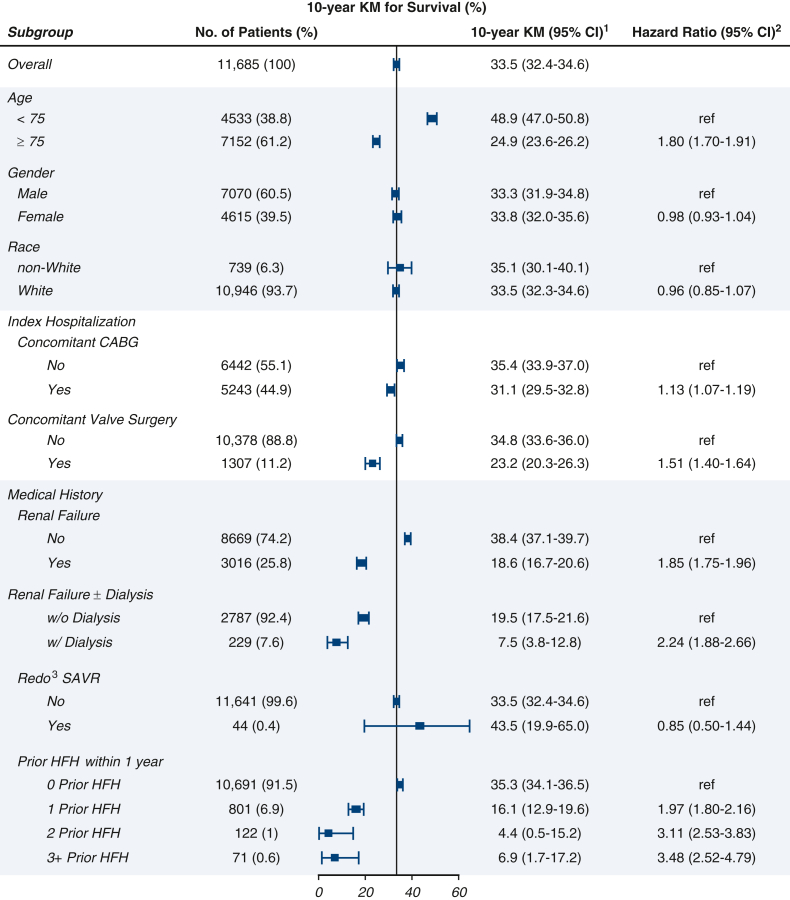
Subgroup analyses for 10-year survival.^1^ Kaplan–Meier for survival.^2^ HR for mortality.^3^ Redo = history of prior TAVI or surgical aortic valve repair/replacement. Vertical line represents the overall 10-year survival (33.5%). *KM*, Kaplan–Meier; *CI*, confidence interval; *CABG*, coronary artery bypass grafting; *SAVR*, surgical aortic valve replacement; *HFH*, heart failure rehospitalization.

**Figure E6:**
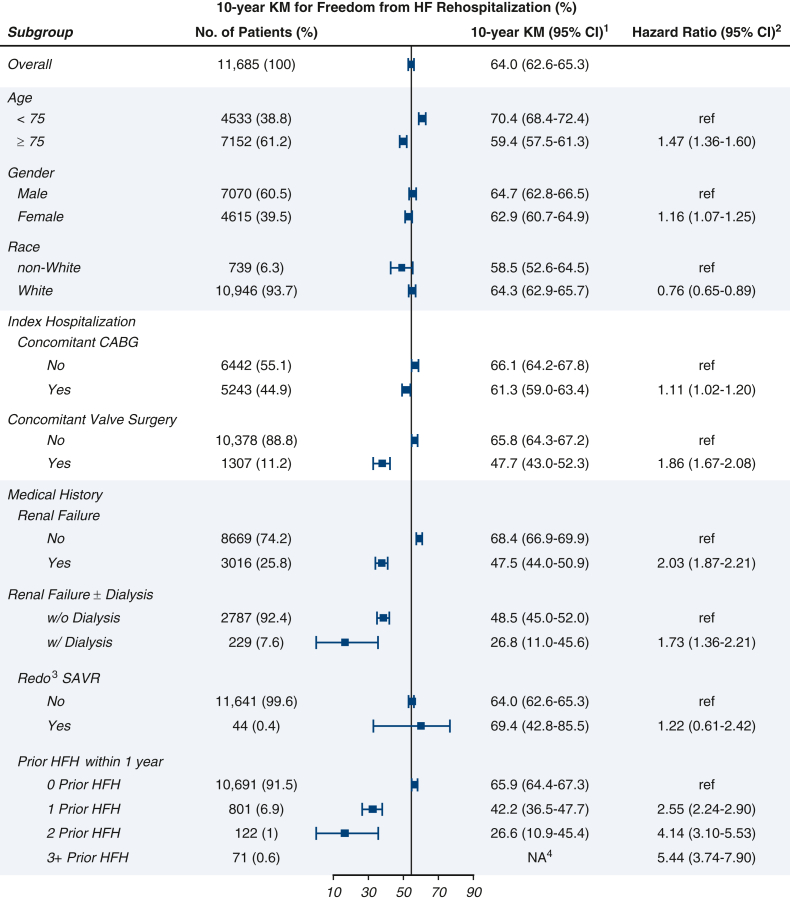
Subgroup analyses for 10-year freedom from HF rehospitalization. ^1^ Kaplan–Meier for freedom of HF rehospitalization.^2^ HR for HF rehospitalization.^3^ Redo = history of prior TAVI or surgical aortic valve repair/replacement.^4^ Not available due to no data available at 10 years. *Vertical line* represents the overall 10-year freedom from HF rehospitalization (64.0%). *KM*, Kaplan–Meier; *HF*, heart failure; *CI*, confidence interval; *CABG*, coronary artery bypass grafting; *SAVR*, surgical aortic valve replacement; *HFH*, heart failure rehospitalization.

The 10-year survivals and hazard ratios (HRs) for all-cause mortality are summarized for various subgroups of interest in Figure 5. Differences in 10-year mortality were observed across subgroups based on age, concomitant surgery, and medical history. Similar results were observed for 10-year HF rehospitalization (Figure E6).

The 10-year survival ranged from 38.4% in patients without renal failure to 19.5% in patients with renal failure without dialysis and 7.5% in patients with renal failure on dialysis. The 1-year survival for isolated SAVR in patients on dialysis was 70.3% (95% CI, 59.7-78.5). Freedom from rehospitalization for HF at 10 years postimplant ranged from 68.4% in patients without renal failure to 26.8% for patients with renal failure requiring dialysis (Figure E6).

### Independent Predictors

Table 3 shows the independent predictors for 10-year all-cause mortality. After adjusting for other covariates, patient age and the presence of concomitant procedures were significantly associated with the 10-year mortality after SAVR. The top 2 independent predictors of the 10-year mortality were baseline renal failure (HR, 1.46; 95% CI, 1.38-1.55) and HF (HR, 1.41; 95% CI, 1.33-1.49). These 2 independent predictors along with performing a concomitant other cardiac valve surgery were among the top 3 predictors of the 10-year HF rehospitalization (renal failure HR, 1.60; 95% CI, 1.46-1.74; HF HR, 1.48; 95% CI, 1.35-1.61; and other cardiac valve surgery HR, 1.54; 95% CI, 1.37-1.73) (Table E6). Patients who underwent a concomitant other cardiac valve surgery had a higher incidence of HF and renal failure preoperatively compared with the rest of the patients, which increases the risk for reduced survival and more HF rehospitalizations (Table E5).

**Table 3:** Predictors for 10-year all-cause mortality

Adverse event	Alive (N = 5791)	Death (N = 5894)	HR (95% CI)	Multivariate *P*∗
Patient demographics				
Age at implant (y)	74.5 ± 6.7	78.1 ± 7.0	1.05 (1.04-1.05)	<.001
Gender (female)	2270 (39.2%)	2345 (39.8%)	0.95 (0.90-1.01)	.087
Race (White)	5389 (93.1%)	5557 (94.3%)	0.94 (0.84-1.05)	.262
Implant characteristics				
Concomitant CABG	2496 (43.1%)	2747 (46.6%)	1.12 (1.07-1.19)	<.001
Other cardiac valve repair or replacement (mitral, tricuspid, or pulmonary)	504 (8.7%)	803 (13.6%)	1.34 (1.24-1.46)	<.001
Patient characteristics				
Renal failure	1110 (19.2%)	1906 (32.3%)	1.46 (1.38-1.55)	<.001
HF	2443 (42.2%)	3586 (60.8%)	1.41 (1.33-1.49)	<.001
Chronic pulmonary disease	2874 (49.6%)	3625 (61.5%)	1.25 (1.18-1.32)	<.001
Diabetes	2594 (44.8%)	3105 (52.7%)	1.20 (1.13-1.26)	<.001
Atrial fibrillation	1562 (27.0%)	2362 (40.1%)	1.19 (1.12-1.26)	<.001
Previous myocardial infarction	1059 (18.3%)	1576 (26.7%)	1.17 (1.10-1.24)	<.001
Coagulopathy	691 (11.9%)	1053 (17.9%)	1.17 (1.09-1.26)	<.001
Liver disease	748 (12.9%)	826 (14.0%)	1.12 (1.03-1.21)	.005
Peripheral vascular disease	3166 (54.7%)	3673 (62.3%)	1.08 (1.02-1.14)	.008
Cerebrovascular disease	3331 (57.5%)	3772 (64.0%)	1.04 (0.98-1.10)	.212
Obesity	1441 (24.9%)	1178 (20.0%)	0.96 (0.90-1.03)	.280
Hypertension	5425 (93.7%)	5622 (95.4%)	0.90 (0.79-1.02)	.093

*HR*, Hazard ratio; *CI*, confidence interval; *CABG*, coronary artery bypass grafting; *HF*, heart failure.

∗ Multivariable Cox regression.

## Discussion

This real-world nationwide study of US Medicare beneficiaries undergoing SAVR with the Epic Supra valve between 2008 and 2019 demonstrates that long-term clinical outcomes are impacted by underlying comorbidities and the need for concomitant cardiac procedures. Unlike prior studies limited to isolated SAVR in the Medicare population,^14^^,^^15^ this study provides clinical outcomes over 10 years of follow-up using a single bioprosthetic valve choice for a broader SAVR population undergoing various combinations of concomitant cardiac procedures. The observed operative mortality through 30 days or during the index hospitalization ranged from 3.1% for isolated SAVR to 24.6% for emergency SAVR with an overall average of 5.7% for the entire population. These outcomes are consistent with data from a recent study by Lauck and colleagues^4^ examining temporal changes in mortality after SAVR and TAVI in a Medicare population, where the 30-day mortality remained relatively unchanged at 4.5% ± 0.2% for SAVR and decreased from 6.3% to 2.0% for TAVI between 2012 and 2019, respectively.^4^ Over the same period, Lauck and colleagues^4^ reported a decrease in hospital length of stay from 12.2 to 10.5 days for SAVR versus from 9.1 to 3.5 days for TAVI, where the reported length of stay for SAVR is consistent with our data.^4^

As previously shown, the presence of baseline HF was a strong influencer for operative mortality, long-term survival, and rehospitalization for HF.^14^^,^^15^ In a study conducted by Vassileva and colleagues^14^ using a Medicare population who underwent isolated SAVR between 2000 and 2009, it was demonstrated that the presence of preoperative HF was associated with an increased operative mortality of 8.5% versus 3.5%. Additionally, it was shown that operative mortality increased from 7.4% to 19.2% depending on the number of preoperative HF hospitalizations. In the same study, it was shown that long-term survival for patients undergoing isolated SAVR was influenced by the presence of preoperative HF, where the survival for patients without preoperative HF at 10 years postimplant versus with preoperative HF aligns with the results of our study (48.3% vs 26.3%, respectively). Our subgroup analyses similarly showed a trend of decreasing 10-year survival as the number of HF rehospitalizations in the year before SAVR increased.

In the study conducted by McNeely and colleagues,^15^ the cumulative incidence of rehospitalization for HF in a Medicare population who underwent isolated SAVR between 2000 and 2004 was 17% at 5 years postimplant. It was also shown that the cumulative incidence of HF rehospitalization increased as the number of HF hospitalizations in the year before SAVR increased. Both of these observations are consistent with our findings.

Preoperative renal failure was also found to be a strong influencer for long-term survival and rehospitalization for HF. Mentias and colleagues^16^ compared clinical outcomes between SAVR and TAVI performed between 2015 and 2017 in Medicare beneficiaries with end-stage renal disease on hemodialysis. The reported 30-day mortality for patients undergoing SAVR was higher than for those undergoing TAVI (12.8% vs 4.6%, *P* < .001). However, this difference subsequently decreased at 1 year postimplant (31.0% vs 28.1%, *P* = .1). In a similar study by Kobrin and colleagues^17^ with procedures performed in 2011 and 2012, there was also no difference in the 1-year survival between SAVR and TAVI (63.6% vs 60.0%, log-rank *P* = .99). A similar survival at 1 year postimplant for patients on dialysis undergoing isolated SAVR was observed in our study.

Patients requiring concomitant procedures such as CABG and other cardiac valve replacement or repair surgery (mitral, tricuspid, or pulmonary) had an increase in operative mortality with a reduced long-term survival and more rehospitalization for HF compared with patients undergoing an isolated SAVR. The addition of CABG resulted in a modest increase in operative mortality, whereas the addition of other cardiac valve surgery had a more substantial increase in operative mortality. Patients requiring additional cardiac valve surgery had a higher incidence of preoperative HF (73.7% vs 49.7%), which may explain the higher observed operative mortality, reduced long-term survival, and long-term increase in HF rehospitalization.

It is anticipated that with TAVI being performed in lower-risk Medicare patients, there will be a corresponding relative decrease in the number of isolated SAVRs being performed over time.^3^ However, because Food and Drug Administration approval for low-risk patients undergoing TAVI did not occur until August 2019, this trend was not observed in our study cohort with implants performed between 2008 and 2019. Surgeons engaged in counseling patients with aortic valve disease who may not be suitable candidates for TAVI and needing to undergo a concomitant cardiac surgery may use the data from our study to inform patients of the anticipated survival and the risk for HF rehospitalization after SAVR. Long-term outcomes are primarily driven by the presence of preoperative HF and renal failure and the need for concomitant procedures, providing potential guidance on clinical decision-making regarding treatment options such as SAVR versus TAVI.

The design of the Epic Supra valve is based on the Biocor valve except for having the Linx anticalcification treatment.^12^ The Biocor valve was first introduced in 1982 and has more than 25 years of proven durability with a freedom from all-cause valve reoperation of 90.6% at 10 years postimplant.^18^ In this real-world nationwide study, the freedom from all-cause valve reintervention at 10 years postimplant was 94.6%, which is consistent with the 97.3% ± 0.5% freedom from reintervention due to structural valve deterioration reported at 10 years after implant from Leipzig University for all patients undergoing SAVR with the Epic valve.^19^ Approximately one-third of the patients undergoing valve reintervention in our study were managed successfully with a transcatheter VIV intervention.

### Study Limitations

The study results are based on site-reported administrative Medicare claims data, in which the derived patient characteristics and procedural data are dependent on the billing codes used to support reimbursement for healthcare services rather than research purposes. Because of inherent limitations within the billing codes, certain parameters, such as the underlying aortic valve disease etiology, left ventricular ejection fraction, and New York Heart Association HF classification status, are unknown. Additionally, at the time of valve reintervention the exact mode of structural valve deterioration is unknown. However, the Medicare claims data have been found to be extremely informative and validated as a useful data source for gaining insights into the clinical outcomes associated with a wide range of procedures including surgical and transcatheter cardiac valve interventions.^20^^,^^21^ Although the Medicare data do not provide New York Heart Association HF functional status, the number of HF hospitalizations in the year before implant may be used as an indicator for HF severity.

Approximately 98% of the United States population aged 65 years or older are covered by Medicare insurance plans.^22^ However, this study is limited to Medicare FFS beneficiaries and does not include patients enrolled in the Medicare Advantage plans due to limitations in data availability. Between 2008 and 2019, FFS Medicare represented 64% to 78% of all Medicare beneficiaries.^23^ Medicare Advantage patients are generally similar to Medicare FFS in terms of demographics, with a recent trend of having younger and more non-White patients.^24^ Although not all Medicare beneficiaries or Epic Supra valve implants were captured in this study, the data from this study represent the population that is most commonly treated with SAVR and are informative to patients contemplating SAVR because the results obtained may be used to predict clinical outcomes depending on individual patient comorbidities, such as HF and renal failure, and the need for any concomitant procedures.

Finally, the findings from this study observed in the Medicare population may not be generalized to younger populations, who may have improved survival with reduced HF hospitalization but with a higher incidence of aortic valve reintervention.^18^ However, a comparison of the baseline demographics from this study cohort with other studies with a larger Medicare cohort^3^^,^^14^^,^^15^ undergoing isolated SAVR shows similar age (76 years), gender (45%-50% female), and race (93% White), such that the results from this study may be generalized to other Medicare patients. It is uncertain whether the results of this study may be generalized to non-White minority patients because 93% of the patients in this study were White. Further, the results from this study are specific to the Epic Supra valve and may not be generalized to SAVR with bioprosthetic valves from other medical device manufacturers.

## Conclusions

This nationwide study reflects the real-world experience of Medicare beneficiaries undergoing SAVR with the Epic Supra valve and demonstrates that clinical outcomes are influenced by underlying comorbidities and the need for concomitant procedures. Renal failure and HF were found to be the 2 top predictors impacting long-term survival and HF rehospitalization. Overall, the Epic Supra valve had excellent durability with more than 94% freedom from all-cause reinterventions at 10 years postimplant and is a suitable choice for SAVR in the Medicare population.

### Conflict of Interest Statement

R.J.W. and L.Y.L. are consultants to Abbott. Y.Y., J.B.P., and D.G. are employees of Abbott. All other authors reported no conflicts of interest.

The *Journal* policy requires editors and reviewers to disclose conflicts of interest and to decline handling or reviewing manuscripts for which they may have a conflict of interest. The editors and reviewers of this article have no conflicts of interest.

## Figures and Tables

**Table E1:** Model numbers for Epic Supra Valve

Valve model number	Tissue annulus diameter
ESP100-19-00	19 mm
ESP100-21-00	21 mm
ESP100-23-00	23 mm
ESP100-25-00	25 mm
ESP100-27-00	27 mm

**Table E2:** Procedure codes for surgical aortic valve replacement

Procedures	ICD-9-PCS	ICD-10-PCS
Open and other replacement of aortic valve with tissue graft	35.21	02RF0[7/8/K]Z, X2RF032
Open and other replacement of aortic valve	35.22∗	
Endovascular replacement of aortic valve	35.05∗	
Transapical replacement of aortic valve	35.06∗	

*ICD-9*, International Classification of Diseases, Ninth Revision; *PCS*, procedure coding system; *ICD-10*, International Classification of Diseases, Tenth Revision.

∗ Due to the nonspecificity of ICD-9-PCS codes for the type of heart valves, additional codes are included in the linkage process to identify all the potential tissue heart valves in CMS claims.

**Table E3:** Diagnosis and procedure codes for comorbidities, medical history, heart failure rehospitalization, and concomitant procedures

Conditions	ICD-9-CM	ICD-10-CM
Endocarditis	996.61, 421.0, 421.9, 421.1, 424.90, 424.99, 424.91	T82.6XXA, I33.0, I33.9, I38, I39
Previous myocardial infarction	410.x, 412.x	I21.x, I22.x, I25.2
Heart failure	398.91, 402.01, 402.11, 402.91, 404.01, 404.03, 404.11, 404.13, 404.91, 404.93, 425.4 - 425.9, 428.x	I09.9, I11.0, I13.0, I13.2, I25.5, I42.0, I42.5 - I42.9, I43.x, I50.x, P29.0
Renal failure	403.01, 403.11, 403.91, 404.02, 404.03, 404.12, 404.13, 404.92, 404.93, 585.x, 586.x, 588.0, V42.0, V45.1, V56.x	N18.x, N19.x, Z49.0x, Z49.3x, Z91.15, Z94.0, Z99.2
Atrial fibrillation	427.31	I48.0, I48.1x, I48.2x, I48.91
Cerebrovascular disease	362.34, 430.x–438.x	G45.x, G46.x, H34.0, I60.x–I69.x
Peripheral vascular disease	093.0, 437.3, 440.x, 441.x, 443.1 - 443.9, 447.1, 557.1, 557.9, V43.4	I70.x, I71.x, I73.1, I73.8, I73.9, I77.1, I79.0, I79.2, K55.1, K55.8, K55.9, Z95.8, Z95.9
Hypertension	401.x, 402.x–405.x	I10.x, I11.x–I13.x, I15.x, O10.x, O11.x, O16.x
Chronic pulmonary disease	416.8, 416.9, 490.x - 505.x, 506.4, 508.1, 508.8	I27.8, I27.9, J40.x - J47.x, J60.x - J67.x, J68.4, J70.1, J70.3
Diabetes	250.0–250.3, 250.4–250.9	E08.0, E08.1, E09.0, E09.1, E09.9, O24.0-O24.3, O24.8-O24.9, E10.0-E10.9, E11.0-E11.9, E12.0-E12.9, E13.0-E13.9, E14.0-E14.9, P70.2
Obesity	278.0	E66.x, O99.2x, R939, Z68.3x-Z68.5x
Liver disease	070.22, 070.23, 070.32, 070.33, 070.44, 070.54, 070.6, 070.9, 456.0–456.2, 570.x, 571.x, 572.2–572.8, 573.3, 573.4, 573.8, 573.9, V42.7	B18.x, I85.x, K70.x, K72.x–K74.x, K76.0, K76.2–K76.9, Z94.4
Coagulopathy	286.x, 287.1, 287.3-287.5	D65-D68.x, D69.1
Cardiogenic shock	785.51	R57.0
Heart failure hospitalization (primary diagnosis only)	402.01, 402.11, 402.91, 404.01, 404.11, 404.91, 404.03, 404.13, 404.93, 428, 428.9, 428.1, 428.2, 428.21, 428.22, 428.23, 428.3, 428.31, 428.32, 428.33, 428.4, 428.41, 428.42, 428.43	I11.0, I13.0, I13.2, I50.9, I50.1, I50.20, I50.21, I50.22, I50.23, I50.30, I50.31, I50.32, I50.33, I50.40, I50.41, I50.42, I50.43

**Procedures**	**ICD-9-PCS**	**ICD-10-PCS**
Dialysis	39.95, 54.98	5A1D[0/6/7-9]0Z, 3E1M39Z
Concomitant procedures		
CABG	36.1x	021x[0/4]xx
Other cardiac valve surgery including surgical valve repair or replacement (mitral/tricuspid/pulmonary)	35.23, 35.24, 35.12, 35.33, 35.25, 35.26, 35.13, 35.27, 35.28, 35.14	02RG0[7/8/J/K]Z, 02QG0Z[E/Z], 02UG0[7/8/J/K][E/Z], 027G0[4/D/Z]Z, 02NG0ZZ, 02RJ0[7/8/J/K]Z, 02QJ0Z[G/Z], 02UJ0[7/8/J/K][G/Z], 027J0[4/D/Z]Z, 02NJ0ZZ, 02RH0[7/8/J/K]Z, 02QH0ZZ, 02UH0[7/8/J/K]Z, 027H0[4/D/Z]Z, 02NH0ZZ
Surgical aortic valve repair	35.11	02QF0Z[J/Z], 02UF0[7/8/J/K][J/Z]
Acute MCS	37.61, 37.68, 39.65	5A02[1/2]10, 5A1522[3/F/G/H], 5A021[1/2][6/D]

All comorbidities were assessed for all available inpatient and outpatient claims before the index SAVR hospitalization (excluding the SAVR admission). From Elixhauser and Charlson comorbidity code algorithms. Coding based on Moore BJ, White S, Washington R, Coenen N, Elixhauser A. Identifying increased risk of readmission and in-hospital mortality using hospital administrative data: The AHRQ Elixhauser Comorbidity index. *Med Care*. 2017;55:698-705 and HCSRN: Health care systems research network http://hcsrn.org/sas/standard_macros.sas; Quan H, Sundararajan V, Halfon P, et al. Coding algorithms for defining comorbidities in ICD-9-CM and ICD-10 administrative data. *Med Care*. 2005;43:1130-9. *ICD-9-CM*, International Classification of Diseases, 9th Revision, Clinical Modification; *PCS*, procedure coding system; *ICD-10-CM*, International Classification of Diseases, 10th Revision, Clinical Modification; *CABG*, coronary artery bypass grafting; *MCS*, mechanical circulatory support.

**Table E4:** Procedure codes for terminal end point and surgical/transcatheter aortic valve reinterventions

Procedures	ICD-9-PCS	ICD-10-PCS
Aortic valve reinterventions∗		
SAVR (THVs or MHVs)	35.21, 35.22	02RF0[7/8/J/K]Z, X2RF032
Transcatheter VIV (TAVI)	35.05, 35.06	02RF3[7/8/J/K][H/Z], 02RF4[7/8/J/K]Z, X2RF[3/4]32
Terminal end point		
LVAD	37.66	02HA0QZ
Heart Transplant	37.51	02YA0Z[0/1/2]

*ICD-9*, International Classification of Diseases, 9th Revision; *PCS*, procedure coding system; *ICD-10*, International Classification of Diseases, 10th Revision; *SAVR*, surgical aortic valve replacement; *THV*, tissue heart valve; *MHV*, mechanical heart valve; *VIV*, valve-in-valve; *TAVI*, transcatheter aortic valve implantation; *LVAD*, left ventricular assist device.

∗ Reinterventions are defined as SAVR with THVs or MHVs, or transcatheter VIV aortic replacement.

**Table E5:** Patient Demographics, medical history, implanted valve size, and clinical outcomes

Variable	Isolated SAVR (N = 5356)	SAVR + CABG (N = 4290)	SAVR + valve (N = 703)	SAVR + CABG + valve (N = 395)	*P* value∗
Demographics					
Age at implant (y)	76.1 ± 7.3	76.8 ± 6.6	75.9 ± 7.6	75.9 ± 6.9	<.001
Gender (female)	2423 (45.2%)	1280 (29.8%)	379 (53.9%)	160 (40.5%)	<.001
Race (White)	4965 (92.7%)	4087 (95.3%)	658 (93.6%)	368 (93.2%)	.001
Medical history					
Renal failure	1256 (23.5%)	1120 (26.1%)	215 (30.6%)	119 (30.1%)	<.001
Atrial fibrillation	1763 (32.9%)	1203 (28.0%)	398 (56.6%)	167 (42.3%)	<.001
HF	2664 (49.7%)	1956 (45.6%)	518 (73.7%)	261 (66.1%)	<.001
Previous myocardial infarction	996 (18.6%)	1067 (24.9%)	141 (20.1%)	113 (28.6%)	<.001
Diabetes	2529 (47.2%)	2179 (50.8%)	301 (42.8%)	199 (50.4%)	<.001
Hypertension	5032 (94.0%)	4088 (95.3%)	666 (94.7%)	364 (92.2%)	.006
Liver disease	780 (14.6%)	511 (11.9%)	99 (14.1%)	44 (11.1%)	.001
Chronic pulmonary disease	2933 (54.8%)	2264 (52.8%)	472 (67.1%)	246 (62.3%)	<.001
Peripheral vascular disease	3109 (58.0%)	2510 (58.5%)	395 (56.2%)	236 (59.7%)	.621
Cerebrovascular disease	3200 (59.7%)	2691 (62.7%)	406 (57.8%)	233 (59.0%)	.006
Coagulopathy	766 (14.3%)	531 (12.4%)	152 (21.6%)	76 (19.2%)	<.001
Obesity	1246 (23.3%)	925 (21.6%)	158 (22.5%)	73 (18.5%)	.057
Year of implant					
Implant year (2008-2009)	1268 (23.7%)	1102 (25.7%)	197 (28.0%)	121 (30.6%)	
Implant year (2010-2011)	1972 (36.8%)	1530 (35.7%)	235 (33.4%)	>150 (>38.0%)	
Implant year (2012-2013)	958 (17.9%)	660 (15.4%)	108 (15.4%)	63 (15.9%)	<.001
Implant year (2014-2015)	623 (11.6%)	492 (11.5%)	90 (12.8%)	35 (8.9%)	
Implant year (2016-2017)	341 (6.4%)	297 (6.9%)	42 (6.0%)	15 (3.8%)	
Implant year (2018-2019)	194 (3.6%)	209 (4.9%)	31 (4.4%)	<11 (<2.8%)	
Implanted valve size, mm					
19	646 (12.1%)	396 (9.2%)	112 (15.9%)	50 (12.7%)	
21	1661 (31.0%)	1182 (27.6%)	226 (32.1%)	119 (30.1%)	
23	1763 (32.9%)	1579 (36.8%)	202 (28.7%)	131 (33.2%)	<.001
25	946 (17.7%)	922 (21.5%)	123 (17.5%)	80 (20.3%)	
27	340 (6.3%)	211 (4.9%)	40 (5.7%)	15 (3.8%)	
Clinical outcomes					
Terminal reintervention (LVAD or heart transplant)	<11	0	0	<11	.320
Follow-up after implant (y)	5.5 (3.2)	5.3 (3.2)	4.7 (3.4)	4.5 (3.4)	<.001

*SAVR*, Surgical aortic valve replacement; *CABG*, coronary artery bypass grafting; *VALVE*, other cardiac valve repair or replacement (mitral, tricuspid, or pulmonary); *HF*, heart failure; *LVAD*, left ventricular assist device.

∗ One-way analysis of variance test for continuous variables, chi-square test for categorical variables.

**Table E6:** Predictors for 10-year heart failure rehospitalization

Adverse event	No HFH (N = 9185)	HFH (N = 2500)	HR (95% CI)	Multivariate *P* value∗
Patient demographics				
Age at implant (y)	76.1 ± 7.0	77.3 ± 7.3	1.03 (1.02-1.03)	<.001
Gender (female)	5643 (61.4%)	1427 (57.1%)	1.15 (1.06-1.25)	.001
Race (White)	8624 (93.9%)	2322 (92.9%)	0.79 (0.68-0.93)	.003
Implant characteristics				
Concomitant CABG	4093 (44.6%)	1150 (46.0%)	1.18 (1.09-1.28)	<.001
Other cardiac valve repair or replacement (mitral, tricuspid, or pulmonary)	919 (10.0%)	388 (15.5%)	1.54 (1.37-1.73)	<.001
Patient characteristics				
HF	4399 (47.9%)	1630 (65.2%)	1.60 (1.46-1.74)	<.001
Renal failure	2160 (23.5%)	856 (34.2%)	1.48 (1.35-1.61)	<.001
Atrial fibrillation	2806 (30.5%)	1118 (44.7%)	1.46 (1.34-1.59)	<.001
Chronic pulmonary disease	4883 (53.2%)	1616 (64.6%)	1.34 (1.23-1.46)	<.001
Diabetes	4275 (46.5%)	1424 (57.0%)	1.33 (1.22-1.44)	<.001
Previous myocardial infarction	1915 (20.8%)	720 (28.8%)	1.25 (1.14-1.37)	<.001
Obesity	1964 (21.4%)	655 (26.2%)	1.25 (1.14-1.37)	<.001
Coagulopathy	1266 (13.8%)	478 (19.1%)	1.17 (1.05-1.30)	.004
Cerebrovascular disease	5492 (59.8%)	1611 (64.4%)	1.05 (0.96-1.14)	.302
Liver disease	1218 (13.3%)	356 (14.2%)	1.03 (0.82-1.15)	.656
Hypertension	8638 (94.0%)	2409 (96.4%)	1.02 (0.82-1.27)	.854
Peripheral vascular disease	5312 (57.8%)	1527 (61.1%)	0.97 (0.89-1.05)	.437

*HFH*, Heart failure hospitalization; *HR*, hazard ratio; *CI*, confidence interval; *CABG*, coronary artery bypass grafting; *HF*, heart failure.

∗ Multivariable Cox regression.
